# HIV-1 Tat-driven Glutamate Dysregulation: Implications for Cognitive Impairment in HAND

**DOI:** 10.1007/s11904-026-00789-w

**Published:** 2026-06-15

**Authors:** Brenna C. Duffy, Michael R. Nonnemacher, Sandhya Kortagere

**Affiliations:** 1 Department of Microbiology and Immunology, Drexel University College of Medicine, Philadelphia, PA USA; 2 Center for Molecular Virology and Translational Neuroscience, Institute for Molecular Medicine and Infectious Disease, Drexel University College of Medicine, Philadelphia, PA USA; 3 Sidney Kimmel Comprehensive Cancer Center, Thomas Jefferson University, Philadelphia, PA 19107 USA

**Keywords:** Astrocytes, Glutamate, HIV-1, HIV-1-associated neurocognitive disorders (HAND), Microglia, Transactivator of transcription (Tat)

## Abstract

**Purpose of Review:**

HIV-1-associated neurocognitive disorders (HAND) manifest in 15% to 50% of people with HIV, impairing learning and memory and executive function. The chronic generation of HIV-1 Transactivator of transcription (Tat) likely contributes to HAND via direct neuronal toxicity and glial-mediated toxicity. This review summarizes our current understanding of how chronic Tat generation from microglia and astrocytes promote glutamate excitotoxicity.

**Recent findings:**

In recent years, the indirect effects of Tat through the activation of glial cells have gained significant interest. This review highlights microglia and astrocytes as HIV-1 reservoirs that release Tat protein in the central nervous system. Specific context is provided on the Tat isoforms and models in the recent literature and their impact on our understanding of the neuronal and glial-mediated effects of Tat on glutamate transmission.

**Summary:**

Transgenic and transduction models of HIV-1 Tat expression in glia have demonstrated Tat-induced glial activation phenotypes that contribute to dysregulation of glutamate receptors and transporters. Investigating both glial-mediated and direct mechanisms of Tat-potentiated excitotoxicity can identify therapeutic targets that are relevant for HAND.

## Introduction

Human immunodeficiency virus type 1 (HIV-1)-associated neurocognitive disorders (HAND) encompass a collection of neurocognitive impairments that vary in severity and functional domain. HIV-1-associated dementia (HAD), which is now largely reduced in prevalence in developed countries, involves motor dysfunction in addition to severe cognitive impairment. In the initial stages of the HIV/AIDS epidemic, HAD and HIV-encephalitis (HIV-E) were more common [1, 2], which contributed heavily to the early literature on the presence of HIV-1 virus and proteins in brain tissue. This review will focus on mechanisms underlying milder forms of HAND, which have become more common in recent years [3]. Asymptomatic neurocognitive impairment (ANI) and mild neurocognitive disease (MND) affect a significant proportion of people living with HIV (PWH) [2]. These forms of HAND involve subtle deficits affecting information processing speed, executive function including learning and memory and decision making [4, 5]. In ANI, symptoms have little impact on day-to-day activities while MND involves a marked impairment of normal function. The long-standing clinical diagnostics for HAND originally focused on distinguishing HAD from milder impairments, and identifying subtypes of HAD in PWH; these criteria were later refined to specify required cognitive domain testing and quantitative criteria for classifying ANI, MND, and HAD [6]. Although the broader clinical guidance for PWH experiencing cognitive impairment is to modify patient anti-retroviral therapy (ART) regimen, that modification has not been shown to be effective for all PWH [7] and may only work on a case-by-case basis. The limited efficacy in this approach leaves a significant population of PWH without relief for cognitive impairment. The continued study of mechanisms underlying HAND is thus crucial for the development of new therapeutic targets, particularly those relevant in chronic infection with viral suppression.

## Viral and Cellular Contributions to Neuropathogenesis

ARTs have drastically reduced the incidence of AIDS, greatly improving the lifespan and quality of life for PWH [2]. However, HIV-1 enters the central nervous system (CNS) early to allow infection of cells in the brain [8, 9]. Previous studies on the pathology of HIV-1 suggested that primary infection affects the CNS [10, 11] but viral replication is readily suppressed by immune infiltration [12]. Nonetheless, primary infection likely mounts inflammatory processes, gliosis [13–16], and white matter changes which may have more long-term impacts on neuropathology [12, 17] despite PWH experiencing few to no detectable neurological symptoms in early infection [13].

Early HIV-1 entry to the CNS [18–20], combined with integration of provirus and delayed diagnosis, facilitate the establishment of viral reservoirs in brain tissue. Microglia are widely accepted as the primary cellular reservoir [21–26] and spread to astrocytes is also likely, based on the presence of astrocytic viral DNA and RNA [21, 24]. HIV-1 DNA was most readily detected in astrocytes in cases of HIV-associated dementia [21, 27]. Examination of HIV-1 proviral distribution in post-mortem tissue indicated that viral load in the CNS varies by brain region, with higher proviral and RNA loads in hippocampus, basal ganglia and temporal lobe [28–30]. As existing available therapies are not curative, provirus persists in viral reservoirs like microglia and astrocytes, enabling viral protein expression [31]. Other glial cells in the brain are not well established as viral reservoirs, but can be affected by viral proteins generated from provirus in microglia and astrocytes. The current landscape of HIV-1 treatment influences a distinct pathogenesis to consider due to the implementation and availability of ART.

### Role of HIV-1 Accessory Proteins in HAND

The available repertoire of ART can inhibit most aspects of the HIV-1 replication cycle. However, PWH often are infected for months to years before diagnosis [32], with recent trends toward increasing late diagnoses [33]. This delay enables transfer of the virus into CNS and integration of viral DNA to establish latent reservoirs. Due to the small size of the HIV-1 genome, provirus is transcribed as a full-length transcript. The numerous splicing acceptor and donor sites enable the generation of various viral transcripts encoding accessory and regulatory proteins [34–36]. Un-spliced transcripts are generated late in the replication cycle, whereas multiply spliced transcripts for *tat*,* rev*, and *nef* are generated early in viral replication [36, 37]. The early abundance of these transcripts makes it likely that Tat, Rev and Nef proteins can be expressed from latent reservoirs in the context of viral suppression. Indeed, various HIV-1 proteins are detectable in PWH. The trans-acting accessory protein Rev has been detected in astrocytes [38] and found to exert toxicity and lethality when administered intracerebroventricularly in mice [39]. The rare detection of Rev in patient samples and lack of mechanism for Rev toxicity, compared to our knowledge of Tat detectability and toxicity, make Rev a less robust contributor to cognitive impairment in HAND particularly in the context of long-term viral suppression. In contrast, the regulatory protein Nef has been detected [40–43], as have anti-Nef antibodies [44]. Nef has also been shown to drive neuronal oxidative stress, suppression of action potentials [45], and neuroinflammation [46], making it a potential source of toxicity in HAND. Nonetheless, Tat has a significant body of literature that demonstrates its relevance in ANI and MND arising in PWH who are virally suppressed and indicates various mechanisms of toxicity.

**Fig. 1:**
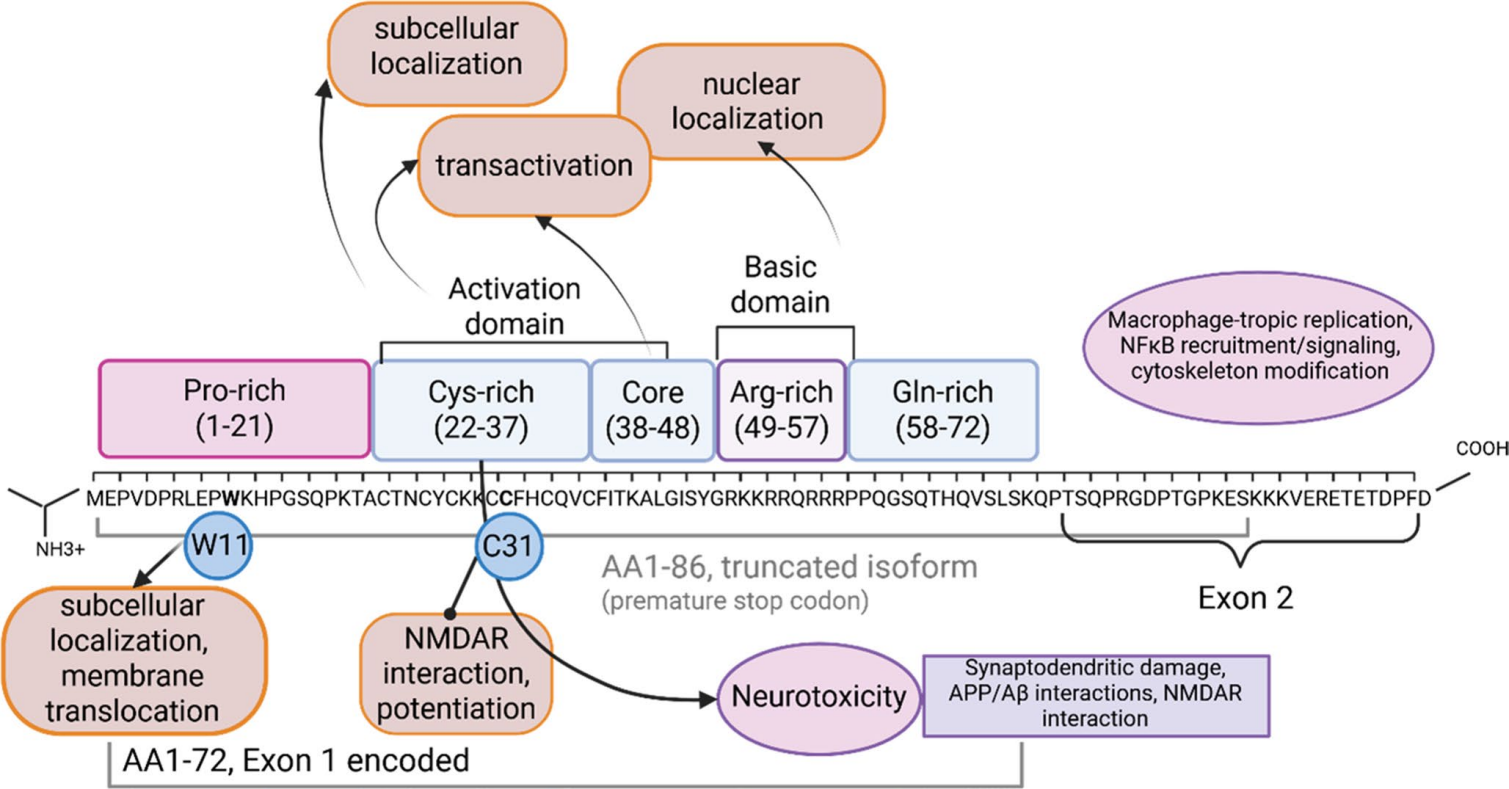
Domains and function of HIV-1 Tat protein (full-length, 101 amino acid clade B). Tat interacts with numerous host cell factors to promote transactivation and viral replication, and to alter cellular function otherwise. Significant residues for neurotoxicity are highlighted in this schematic. The arginine-rich basic domain enables nuclear localization and cellular penetration. The cysteine-rich domain (Cys-rich) is understood to play a central role in neurotoxicity, particularly via residue C31 and its interaction with the NMDA receptor (NMDAR)

Similar to Nef, Tat is an early expressed regulatory protein. Tat regulates viral transcription, splicing regulation, anti-termination and replication [47–50]. Importantly, Tat is detectable in the cerebrospinal fluid (CSF) of PWH on ART [51, 52], and release of Tat is unaffected by ART [53]. Numerous studies suggest that Tat exerts both direct and indirect neurotoxicity, based on the presence of intracellular and extracellular Tat. Thus, both Nef and Tat have clear effects on neuropathogenesis [45, 54–57], however Tat has been more heavily examined and its persistence in CSF makes it a tangible target for study of HIV-mediated neurotoxicity [52, 53]. The persistence of Tat in context of viral suppression, along with evidence of Tat-induced neuronal dysregulation and cognitive impairment in animal models [54, 58–60] posits Tat as a central factor in HAND pathogenesis. Tat is an intrinsically disordered protein [61] and is suggested to alter its conformation when in complex with different host or viral molecules [62, 63]. Tat contains various protein domains that contribute to its’ various functions including viral transcription, protein recruitment, neurotoxicity, and toxicity in general [64–67]; [68–71] (Fig. 1). Specific Tat interactions affecting glutamatergic transmission will be reviewed in later sections, as factors in Tat-mediated mechanisms of HAND.

## Microglia and Astrocytes as Tat Sources

HIV-1 entry to microglia occurs by recognition and binding of CD4 [72] and the CCR5 co-receptor [73–75]. Following viral entry, microglia allow productive infection [76]. Poor penetration of the CNS by existing ARTs allows sustained HIV-1 replication in microglia with intact provirus [25], and current ARTs do not address latently infected cells thus leaving a persistent microglial reservoir in the CNS. This low level of productive infection can spread infection to astrocytes. HIV-1 entry to astrocytes occurs independent of CD4, and in some cases independent of CCR5 and CXCR4, instead binding to the mannose receptor [77]. HIV-1 has been found to infect a small population of astrocytes [78], which may be limited by endocytic uptake leading to lysosomal degradation. In contrast, productive infection and integration in astrocytes occurs via proximity to infected lymphocytes [79]. Ultimately, in PWH on ART, initial infiltration of infected immune cells to the CNS would lead to microglial infection and enable spread of infection to a small population of astrocytes [22]. Based on the detection of viral DNA in astrocytes [21, 27, 80], this spread likely occurs by cell-associated virus rather than cell-free internalization, enabling generation of viral proteins like Tat. Tat specifically has been detected in patient CSF and thus is likely released from latently infected cells either through low viral replication or from defective provirus. Generation of Tat protein by latently infected cells, combined with circulation in EVs and CSF, implies the ability of Tat to circulate and impact uninfected cells; thus, glial cells can act as both sources and ‘targets’ of Tat protein.

## Sources and Distribution of HIV-1 Tat

The mechanism of Tat secretion from cellular reservoirs is still only partially understood. Tat lacks a signal peptide, thus evading shuttling through the endoplasmic reticulum and vesicular trafficking. The tryptophan residue at position 11 (Trp11) and the basic domain are thought to enable membrane binding [81], though the RKK motif at position 49–51 also likely contributes [82]. It has been proposed that Tat may cross the plasma membrane by Tat-mediated membrane pore formation [83], spontaneous translocation, and exosomal release [84]. Tat may also be released in exosomes and EVs, as shown in samples from PWH and in preclinical studies [85–87]. Nonetheless, the pathways by which Tat is released are unclear, and there is poor definition of the cellular population that contributes to release of extracellular Tat. Since Tat has been detected in peripheral blood and CSF of PWH who are virally suppressed [51–53], cellular reservoirs throughout the body are likely generating Tat. Within the CNS, small populations of microglia and astrocytes are likely sources of Tat.

## CNS Cells as Targets of HIV-1 Tat

### Models of Tat-induced Neurotoxicity

Numerous models have now been established to study the neurotoxic effects of Tat expression and exposure (Table 1). This section will review Tat expression and treatment experiments involving microglia, astrocytes and neurons; nonetheless, other cells of the CNS may be affected. Initial studies relied on the original synthesis and purification of Tat (HXB2 strain), which was validated for TAR binding and LTR transactivation [88]. Subsequent studies identified truncated peptides [89] and mutants that enabled the discovery of distinct epitopes required for transactivation and neurotoxic mechanisms [90]. Various in vitro and ex vivo studies utilize recombinant Tat protein to investigate molecular and cellular effects of Tat treatment. Transfection and transduction models have become more common in vitro and provide advantages including improved modeling of Tat generation from infected cells and distinction of direct toxicity from indirect glial-mediated Tat toxicity. In vivo studies similarly use recombinant Tat protein injection to distinct brain regions or treatment of tissue ex vivo, as well as infection (EcoHIV) and transgenic models. Transgenic models have become the predominant approach for evaluating Tat-mediated cognitive impairment. The inducible Tat transgenic mouse model (iTat) [54], in which mice are induced by Doxycycline (Dox) to express Tat86 under the GFAP promoter, has demonstrated various cognitive and behavioral effects of Tat expression throughout the brain. Characterization of the iTat model established that Tat expression leads to BBB dysfunction [91], immune cell infiltration and activation, neurotoxicity [54], and dendritic spine loss throughout the hippocampus [92]. Among all of these models, the Tat86 isoform has been utilized most often to examine Tat-induced toxicity [83]. Few studies have introduced the 101-amino acid isoform (Tat101), which is more commonly detected in PWH [83, 93]. Increasing utilization of the Tat101 isoform (Table 1), adds context for direct neurotoxicity in neuronal cultures as well as mechanisms of glial-mediated toxicity. The models used in different studies will be indicated in subsequent sections to highlight the overall functional effects of Tat toxicity on glutamatergic neurotransmission.

**Table 1:** Tat neurotoxicity literature by isoform and approach

Isoform	Model, cell type if applicable	Outcome	Reference (PMID)
Tat72 (1-100µM; 500nM), Tat86, Tat101 (100ng/ml recombinant or transfection)	Human neurons, rat hippocampal neurons, iTat mice (7d induction)	Direct toxicity - neuron firing and excitability	[94–97]
Tat72 (400nM), Tat31-61 (17 µM, 100 µM)	primary rat hippocampal cultures, human neurons	Direct neurotoxicity	[98, 99]
**Recombinant Tat72** (10-100nM; 10ng/ml; 2pmol, 125 and 700nM; 125nM, 200fmol; 0.8fmol; 200 ng/ml), **Tat86** (0.1-100nM, 1 µM; 12 fmol; 0.32nmol; 50ng/ml)	primary rat hippocampal neurons or slice, rat synaptosomes; human mixed neuronal/glial cultures, SH-SY5Y, primary human neurons	Direct neurotoxicity - via NMDAR	[100–111]
Recombinant Tat (100-500ng), Tat72 (0.5µL of 500pM)	Hippocampal, striatal injection	Direct neurotoxicity - via NMDAR, in vivo	[112, 113]
Recombinant Tat (200 ng/ml; 1-3nM; 100 ng/ml), Tat86 treatment (100nM) or expression, Tat101 expression (primary mouse astrocytes)	SH-SY5Y, SK-N-MC neurons, systemic Tat expression in mice, human and mouse synaptosomes, primary murine astrocytes	Elevated extracellular glutamate	[103, 114–118]
Recombinant Tat86 (1nM), fragment peptides (1-3nM)	Human and rat cortical synaptosomes	Glutamate receptor interactions, neurotransmitter release	[115, 119, 120]
Tat86 expression (iTat uninduced; induced)	Tat transgenic mice (aged uninduced; induced)	Gross pathology, astrogliosis, synaptic damage, Neuroinflammation	[54, 121]
Tat72 (10ng/ml)	Primary human mixed neural cultures	Indirect neurotoxicity - via NMDAR	[101]
Tat86 expression (transduction, transfection)	iTat transgenic primary astrocytes; primary neurons; U373MG	Indirect toxicity, astrocytic glutamate uptake	[122, 123]
Recombinant Tat101, Tat86; ‘full-length’ Tat or Tat clade B expression; Tat86 expression	Human astrocytes, human and rat brain tissue	Indirect toxicity, EAAT2	[124–126]
Tat86 transfection; recombinant Tat101 (200ng/ml), Tat86, Tat72 (100nM); MHC-II restricted Tat transgenic mice	U373MG, primary rat microglia	Indirect toxicity, xCT	[127–130]
**Tat86** (treatment 100ng/mL, transduction, transfection); **Tat101** (treatment 50ng/ml, transfection, transduction – primary mouse astrocytes; 3.57nM – EV induction)	mouse, rat and human astrocytic cultures; rat dentate gyrus injection	Indirect toxicity, astrocytes	[85, 117, 131, 132]
**Tat86** (transfection; treatment 6.95, 35.0, and 69.5 pmol), **Tat72** (50-100nM; 100ng/ml), **Tat101** (50ng/ml)	U87MG and SK-N-MC, U373MG, N9 murine microglia and rat primary microglia, primary human astrocytes	Indirect toxicity - Neuroinflammation	[133–136]
Recombinant Tat86 (500nM)	SK-N-MC, fetal human neurons	Neuroinflammation	[97]
Tat86 (iTat, 2wks induction)	inducible Tat transgenic (iTat) mice	Motor deficits, hyperexcitability	[137]
Tat86 (10nM)	Rat cortical neurons	Neuronal depolarization and excitability	[138]
Tat86 (iTat)	iTat mice, uninduced (young and aged)	Neurotransmitter/metabolite levels	[139]
Tat86 (iTat)	iTat mice	Neurotransmitter/metabolite levels	[140]
Tat86 (40ng), Tat101 (40ng)	in vivo, rat mPFC injection	NMDAR expression	[59]
Recombinant Tat86 10-100nM (Clade B)	Primary medium spiny neurons (murine), WT or MOR KO;	NMDAR mediated toxicity, dendritic damage - striatum/MSNs	[141]
Tat86 expression (iTat), **Recombinant Tat101** (3.57nM – EV induction), **Tat72** (5.7nmol), Systemic Tat transgenic mice	iTat mice, human astrocyte-derived EVs, mice, ventricular (ICV) injection (rat)	Disrupted E: I balance	[85, 116, 142, 143]
**Recombinant Tat86** clade B (50ng/ml; 100ng ICV)	Primary rat hippocampal neurons, ICV injection (mice)	Synaptic loss - via NMDAR	[55, 144, 145]
Tat86 expression (iTat aged, uninduced; iTat induced); recombinant Tat86 (50 ng/ml)	inducible Tat transgenic (iTat) mice, rat hippocampal cultures	Dendritic spine and synapse damage	[54, 60, 92, 110, 121, 142]
Recombinant Tat86 (10ng/ml); Tat101 expression (Transfection), Tat72 (100, 300 ng/ml)	U87MG, U251MG, primary astrocytes	Astrocyte activation	[146, 147]
Tat86 (iTat)	iTat mice	Cognitive impairment, reversal learning	[140, 148]
**Recombinant Tat72** (0.5µL of 500pM), **Tat86** (100ng ICV)	intra-hippocampal (CA1), ICV injection (mice)	Cognitive impairment - NMDAR mediated	[113, 145]
Tat86 (iTat)	iTat mice	Cognitive impairment, Reward processing	[149]
Tat86 (iTat), **Recombinant Tat72** (0.5µL of 500pM; 25 µg) or **Tat86** (0.32nmol, 40ng,10 µg; 5–50 µg in neonatal rats; 1 µM ex vivo)	Tat transgenic mice, hippocampal injection, ventricular injection	Cognitive impairment, spatial learning and memory	[59, 60, 109, 113, 148, 150–153]
Tat86 (iTat)	Tat transgenic mice	Motor impairment	[151]
Tat86 (iTat)	Tat86 (iTat mice), primary murine cultures	Oligodendrocyte impairment (NMDAR mediated)	[154]

### Glutamatergic Mechanisms of HAND

**Fig. 2:**
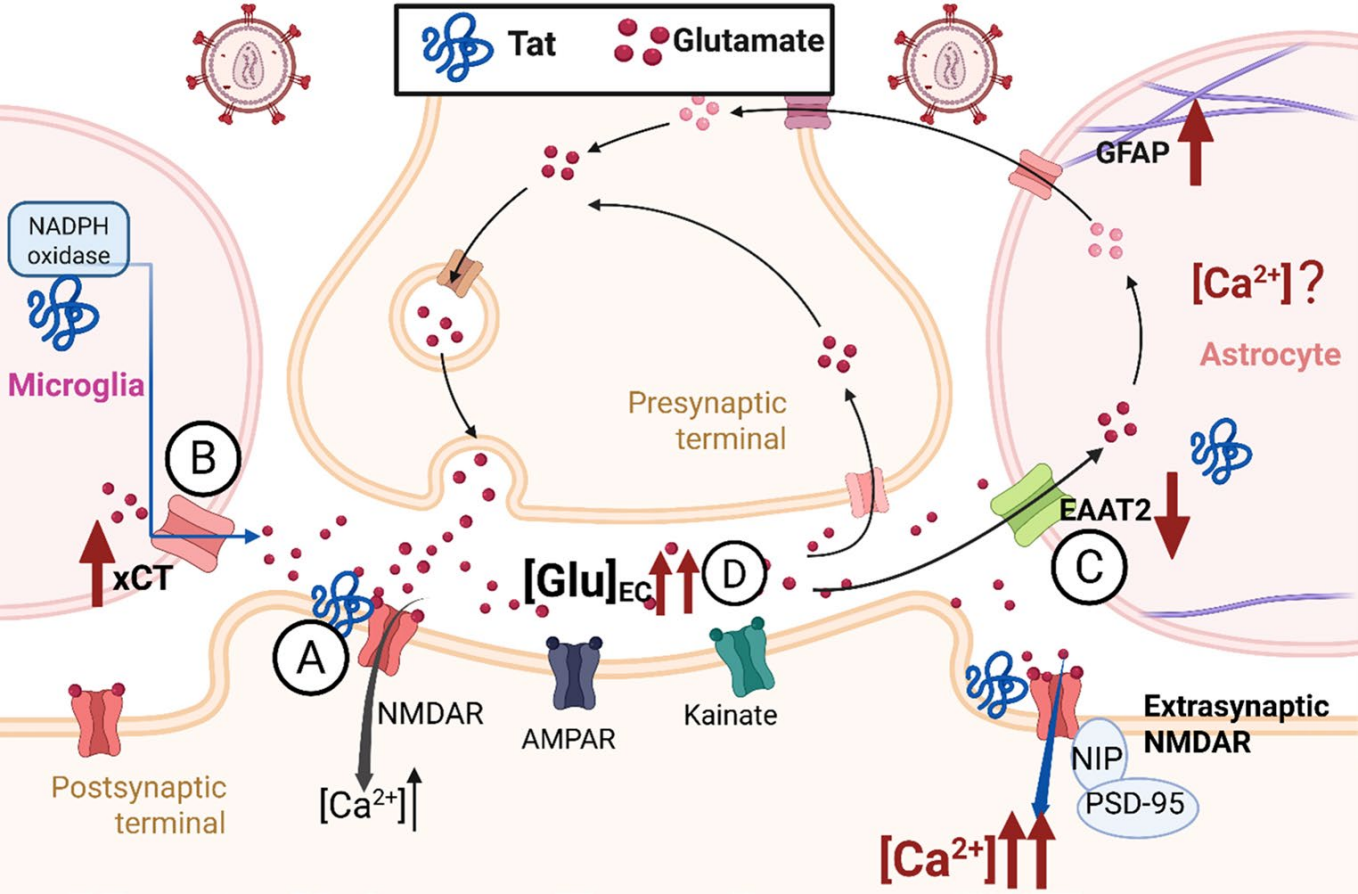
Expression of HIV-1 Tat from viral reservoirs mediates glutamate toxicity. Tat can be expressed intracellularly in a small population of microglia (left), and a smaller population of astrocytes (right); Tat can also be released (by secretion, membrane translocation, or other non-canonical mechanisms) as extracellular protein which may exert direct toxicity at the NMDA receptor (A), or indirect toxicity via pathological xCT (cystine-glutamate antiporter) activity (B) or reduced EAAT2 (excitatory amino acid transporter 2) expression/function (C). Various studies have demonstrated that low concentrations of Tat likely drive sublethal alterations in synapses or dendritic spines, as well as NMDAR composition to cause changes in behavior/cognitive functions. The indirect toxicity exerted via xCT or EAAT2 contributes to overall elevation of extracellular glutamate (D), which can enhance toxicity via extrasynaptic NMDAR, as well as additional effects on astrocytic functions

One proposed central mechanism underlying HAND is Tat-mediated stimulation of excessive excitatory signaling through glutamate [155]. Executive functions affected in ANI and MND require appropriate glutamatergic tone and circuitry involving multiple brain regions (prefrontal cortex, hippocampus, and basal ganglia), further emphasizing its importance in HIV-1-mediated cognitive impairment. Glutamate toxicity is currently accepted as a mechanism involved in various neurological diseases (Alzheimer’s, Parkinson’s, etc.), making it a robust area for novel therapeutic development. Other HIV-1 proteins, and HIV-1 infection, are also suggested to dysregulate glutamate homeostasis [123, 156, 157], emphasizing this as a likely central mechanism in HAND – as the effects of multiple viral proteins alongside other mechanisms could have combined effects on glutamatergic tone and transmission. Tat’s numerous binding partners, along with its persistence in PWH on ART, provides a robust system to assess the involvement of glutamate toxicity in HAND. Tat protein has numerous mechanisms by which it interacts with glutamatergic transmission, including receptor and transporter interactions that may have synergistic effects on cognition (Fig. 2). The study of HIV-1 Tat as a neurotoxin thus provides a strong opportunity to identify mechanisms of glutamate toxicity in HAND and potential therapeutic targets.

### Tat-induced Neuronal Toxicity

Recombinant Tat protein was identified as the only neurotoxic species when tested alongside precursor gp160-, p25-, and nef-derived peptides [158], demonstrating Tat’s specific binding to synaptosomes as well as mouse neuroblastoma and rat glioma cells, and toxicity on rat glioma cells. Additionally, Tat86 peptides depolarized neurons and depressed membrane resistance in multiple models [158]. The mechanism by which Tat depolarizes neurons and causes neurotoxicity was further characterized with additional peptides and mutants. Truncation to Tat72 (HIV-1 BRU) severely reduced Tat uptake [89], whereas Tat86 taken into cells was predominantly localized to the nucleus [89]. These early findings emphasized the importance of residues 1–86 for optimal cellular uptake, and the ability of extracellular Tat to reach the nucleus whether to transactivate HIV-1 provirus in infected cells or alter gene expression in bystander cells.

Numerous studies utilizing recombinant Tat have established that specific epitopes interact with glutamate receptors to enable neurotoxicity. An early study identified that low dose (100ng) recombinant Tat in neonatal rat hippocampus and striatum enhanced NMDA-induced neurotoxicity [112]. Glutamate receptor-dependent neurotoxicity was verified in human neurons treated with low millimolar concentrations of Tat31-61 peptide, as well as SK-N-MC and fetal human neuron cultures treated with Tat86 [97]. Tat-induced apoptosis in fetal neurons was opposed by AMPA receptor antagonist CNQX but not NMDAR antagonists, suggesting that direct Tat toxicity on neurons may be mediated by AMPAR [97] – in contrast to other studies of Tat72 and Tat86 toxicity through interactions with NMDAR. Tat-induced depolarization via glutamate receptors is supported by ex vivo assessment in rat brain slices indicating that non-NMDA receptors are involved in Tat-mediated enhancement of neuronal calcium flux, and depolarization of CA1 pyramidal neurons, via residues in the 33-47AA peptide [99]. Recombinant Tat30-86 peptide as well as Tat72 calcium-dependently depolarize rat neurons in culture and ex vivo tissue preparations as well as human neuron cultures [94, 138]. The effects of these various peptides likely involve the C30-C31 motif which is now known to mediate Tat interaction with the NMDAR [64] (Fig. 1). Tat-induced depolarization is supported by reduction in membrane resistance, increased frequency and amplitude of miniature inhibitory post-synaptic currents (mIPSC) plus increased frequency of miniature excitatory post-synaptic currents (mEPSCs) [138]. Nonetheless, Tat may have heterogeneous effects on excitability, as Tat86 expression in vivo has been shown to increase the intrinsic excitability of medial PFC (mPFC) neurons while decreasing excitability of pyramidal neurons in the CA1 subregion of hippocampus [95]. In concordance with reduced excitability in hippocampus, with Tat101 treatment and expression in hippocampal cultures have shown that Tat reduced neuronal firing [96]. Tat additionally modulated the effects of cocaine, norepinephrine and acetylcholine on neuronal firing by altering GABAergic transmission [96], emphasizing the need to consider both regional and circuit-level effects of Tat through ex vivo and in vivo studies.

The synaptic effects of Tat activity at glutamate receptors also exhibit heterogeneity. Tat86 applied to human and rat synaptosome preparations induced Ca^2+^-dependent acetylcholine (ACh) release through agonism at group I metabotropic glutamate receptors (mGluR) [119]. Tat interaction with mGluR1 has also been found to potentiate NMDA-induced norepinephrine release in human and rat synaptosomes [120]. Further, Tat has been shown to enhance evoked glutamate release from cortical neurons via mGluR1 [115]. Tat86 interaction with mGluRs also enhanced glutamate release in human and mouse neocortical synaptosomes while suppressing GABA release [115]. Tat’s effects on glutamate and GABA release were preserved with Tat37-72 peptide but not with Tat78-86 [115], reinforcing that exon 1-encoded residues are critical for Tat’s induction of glutamate toxicity. These combined effects on glutamate and GABA could alter excitatory/inhibitory balance, enabling diverse cognitive effects by modulating the release of various neurotransmitters important for PFC-mediated functions.

Further investigation of Tat’s potentiation of glutamate signaling led to identification of Tat’s interaction with the NMDAR. Early studies demonstrated that Tat72 potentiated glutamate-induced apoptosis in rat and human neuronal cultures at low doses (10nM, 100nM) [104, 108]. Both pre-exposure and acute exposure to Tat also led to prolonged enhancement of glutamate-dependent and NMDA-induced intraneuronal calcium [108]. In rat cerebral cortical neuron cultures, Tat30-86 induced multiple patterns of intracellular calcium flux, indicating heterogeneous responses to Tat in the cortex [138]. Tat induction of neuronal calcium influx has also been demonstrated in rat hippocampal slice cultures [100], , rat hippocampal and cortical neurons [111] and demonstrated partial dependence on NMDARs [100]. In neuronal cultures, 50nM and 100nM doses of Tat enhanced glutamate-induced neurotoxicity [100, 111], implicating the importance of the NMDAR polyamine site for Tat-induced calcium influx [100]. Direct NMDAR-mediated Tat toxicity has also been observed in SH-SY5Y cultures resulting in activated Caspase-3 and enhanced extracellular glutamate [103]. The interaction of Tat with NMDAR and AMPAR was also found to drive intracellular calcium and sodium flux, and mitochondrial membrane potential in medium spiny neuron dendrites ex vivo [141]. In addition to direct effects on neuronal signaling, Tat’s interaction with glutamate has been shown to induce calcium flux, cell death, and membrane area loss in oligodendrocytes [154]. Thus, Tat is well understood to drive toxicity in both neurons and glial cells via glutamate, with the NMDAR playing a central role.

The mechanism and interactions by which Tat potentiates the NMDAR was elucidated by multiple in vitro and ex vivo studies. Treatment of cortical neurons with Tat induced phosphorylation of NR2A and 2B subunits of NMDA receptor [108]. Evidence of Mg^2+^-, ketamine- and Zn^2+^-sensitive depolarization of hippocampal neurons suggested Tat may interact specifically with the NR1 subunit [105] to induce depolarization. Notably, Tat86 alone has been shown to reverse Zn^2+^ inhibition of the NMDA current mediated by NR1/NR2B recombinant receptors [106], enabling enhancement of NMDA-stimulated currents for 15 min and blocking Zn^2+^ protection against NMDA toxicity [106]. Downstream of the Tat-NMDAR interaction, experiments utilizing recombinant Tat72 induced intracellular Ca2 + in neurons and astrocytes within fetal human cell cultures, with modulation of secondary calcium transients suggesting that Tat induces NMDAR-mediated calcium flux via IP_3_ [102]. These glutamatergic modes of Tat-induced toxicity likely contribute to gross abnormalities. Intraventricular injection of Tat72 (HIV-1 BRU) caused ventricular enlargement, gliosis (enhanced label for microglia and astrocytes via histology), apoptosis and decreased GABA contributing to an enhanced Glutamate to GABA ratio specifically in the hippocampus [143]. These results, while restricted to the hippocampus, emphasize that Tat potentiation of the NMDAR likely contributes to gross abnormalities via excessive glutamatergic signaling.

Beyond the physical interaction with NMDAR and other glutamate receptors, immune and pro-inflammatory factors play an additional role in glutamate toxicity. Tat86 treatment induced TNFα-mediated apoptosis in primary rat neurons, fetal human neurons and SK-N-MC neuroblastoma, independent of NFκB activation [97]. Prior studies have also clearly demonstrated that Tat and TNFα synergistically enhance neuronal apoptosis [159]. Since Tat86 and Tat72) expression and treatment in mouse and rat microglia and astrocyte cultures stimulates TNFα and other pro-inflammatory cytokines [133, 134], these studies together highlight the compounding indirect Tat toxicity via glial-mounted inflammation. In contrast, co-exposure to RANTES (CCL5) or MCP-1 mitigates Tat72-induced neuronal and astrocytic apoptosis in fetal human mixed cultures (30–40% neurons, 60–70% astrocytes, 2–3% microglia) [101]. NMDA induced apoptosis to a similar extent as Tat72, which was also mitigated by RANTES and MCP-1. Further experiments reiterated that NMDA- and Tat-induced neuronal and astrocytic death and increased extracellular glutamate could be limited by MCP-1 and rescued by NMDAR antagonist (MK801) treatment. Further examination indicated that MCP-1 rescues upregulation of NMDAR1 and cell death (both neuron and astrocyte) in Tat72-treated mixed cultures. Interestingly, Tat72 did not induce significant apoptosis in astrocyte-enriched cultures [101], implying that neurons mediate Tat-induced astrocyte toxicity. Thus, Tat induction of cytokine and chemokine responses can potently drive neuronal and glial toxicity in a bidirectional manner.

The collection of findings utilizing recombinant Tat72 suggest that, even with limited cellular uptake of Tat, mechanisms of neuroinflammation and enhanced glutamatergic tone are maintained – such that bystander cells (which are not latently infected with provirus) can initiate and maintain a cascade of sustained inflammation and neurotoxicity. Nonetheless, some of these studies utilize rather high doses of Tat, and Tat72 is not fully representative of Tat detected in PWH on ART [83, 93, 160]. Other studies instead utilize Tat86, which retains neurotoxic effects [89]. Additional studies may still be useful to confirm (A) whether the Tat101 isoform maintains these effects, and (B) more rigorous evidence of physical interaction between Tat and NMDAR (e.g. co-IP, Y2H, FRET, PLA).

Studies of mixed neuronal and glial cultures exposed to Tat provide additional context for the potential indirect modes of Tat toxicity. Tat86-exposed murine striatal cultures (mixed neuronal and glial) contain significantly altered proportions of GFAP + and Iba1 + cells, suggesting Tat exposure reduces astrocyte population while increasing the microglial population [114]. While this study focuses on synergistic effects of Tat and morphine, the data suggests that Tat can stimulate both direct and indirect neurotoxicity, by modulating glial phenotypes and survival [114].

### Tat-mediated Glial Dysregulation and Indirect Neurotoxicity

Tat’s indirect effects on glutamatergic neuronal transmission are supported by Tat-induced glial dysregulation, involving glutamatergic receptors and transporters and innate immune effectors. Notably, Tat has been found to alter microglial glutamate homeostasis and astrocytic glutamate-induced signaling. Astrocytic cultures (rat and human) treated with Tat86 (10 ng/mL, 1 h) or transfected to express Tat (86 or 101aa) exhibited limited glutamate-induced Ca^2+^ flux relative to control conditions [131]. Co-exposure to Tat and TNFα [131] suggested that chronic neuroinflammation and Tat may synergistically alter astrocytic responses to glutamate. Reduced Ca^2+^ flux was dependent on transactivation and active translation, suggesting that Tat induces glutamate insensitivity through an intracellular mediator. Overall, Tat dampened astrocytic responses to glutamate in contrast with the findings in neurons. This is meaningful in the context of the glial HIV-1 reservoir and how released Tat affects distinct cell types. Both neurons and astrocytes express NMDARs, enabling extracellular Tat activity at the receptor; whereas intracellular expression of Tat in astrocytes and microglia may have distinct effects. The dampening of the glutamate-mediated Ca^2+^ response by Tat in astrocytes could result in poor homeostatic function in astrocytes, exacerbating direct effects on neuronal NMDAR potentiation.

The direct effects of Tat on neuronal glutamate signaling are also compounded by cytokine and chemokine release. Such indirect glutamatergic mechanisms of Tat-mediated toxicity have been demonstrated with both Tat72 and Tat86 [101, 114]. Cultures treated with Tat86 and Tat72 demonstrated that Tat can stimulate both direct and glial-mediated neurotoxicity by reducing glutamate buffering in striatal glial cultures without driving apoptosis in astrocytes [114]. In contrast to Tat86, Tat72 (10ng/mL) induced astrocytic apoptosis, enhanced extracellular glutamate and elevated NR1 expression, which were limited by CCL2 and CCL5 [101]. Slightly higher doses of Tat72 (100 ng/ml) have also been shown to induce NF-κB dependent secretion of IL-1β, IL-6, and TNFα in monocytes, and IL-6 upregulation in astrocytes [135]. Importantly, Tat-induced cytokine release may co-occur with enhanced microglial phagocytosis contributing to axonal damage [161], emphasizing the importance of indirect neuronal toxicity via glial cells, and the interaction of glial activation-associated chemokines with glutamate-mediated neurotoxicity. Notably, the contrasting results with Tat72 and Tat86 may be reflective of the importance of residues beyond exon 1 to modulate NF-κB signaling and in turn, indirect Tat neurotoxicity.

More recent studies have elucidated glial-specific mechanisms by which Tat induces glial-mediated glutamate toxicity (Fig. 2). Tat exposure is known to stimulate microglial expression of pro-inflammatory cytokines [134, 136] and upregulate the cystine-glutamate antiporter (xCT) in microglia [129], causing pathological glutamate release [129] (Fig. 2B). This Tat-induced inflammation may contribute to glutamate toxicity in neurons [97], contributing further to a feedback loop between glutamatergic dysregulation and neuroinflammation. The excitatory amino acid transporter 2 (EAAT2 or GLT-1) expressed mainly by astrocytes is additionally downregulated in HIV and SIV infected brain tissue, and Tat expressing astrocytic cultures [123]. EAAT2, which enables rapid reuptake of synaptic glutamate is downregulated in Tat-expressing astrocytes [123]. Non-specific methods of activating EAAT2 have previously been shown to oppose Tat-induced toxicity (loss of viability and mitochondrial toxicity) in human mixed neuronal cultures [162], reinforcing the idea that Tat toxicity involves impaired EAAT2 activity. Additionally, astrocytes express NMDARs [163] and extracellular glutamate stimulates sodium-calcium exchanger (NCX) reverse activity [164] to increase calcium levels in astrocytes. These interactions converge at postsynaptic neurons, where increased extracellular glutamate can activate synaptic and extrasynaptic NMDARs. Alongside direct potentiation of NMDAR by Tat [105, 108], these mechanisms can drive enhanced extracellular glutamate and disrupt the ability for glia to moderate synaptic activity. Nonetheless, these mechanisms have been demonstrated with varying techniques and agnostic to how Tat expression within cellular reservoirs affects Tat-mediated toxicity and cognitive symptoms of HAND.

### Transfection/Transduction Models in Glia

While recombinant protein can enable our understanding of toxicity mediated by extracellular Tat, cellular reservoirs like microglia and astrocytes may be dysregulated distinctly by Tat expression. Transfection and transduction models for Tat expression have improved modeling of intracellular Tat expression in HIV-1 cellular reservoirs. Studies utilizing U373MG astroglial cells demonstrated that Tat86 expression induced oxidative stress, DNA damage, nuclear Nrf2 and xCT upregulation which mediated neurotoxicity in co-cultured neurons [127, 128]. Additionally, either Tat86 treatment or Tat86 expression in U373MG cultures induced has been shown to induce generation of antioxidant molecules [128]. Additional studies of Tat86 expression in U373MG demonstrated upregulation of GFAP protein levels unrelated to proliferation [122], suggesting an activation phenotype [122]. Increase of GFAP promoter activity was also demonstrated in primary iTat astrocytes and coincided with a deficiency in glutamate uptake. Despite the issues of U373MG identity, Tat86 induction of glial activation and indirect neurotoxicity is supported by findings in astrocytic cultures. In U87MG, U251MG and fetal human progenitor-cell derived astrocytes (PDAs) Tat101 expression or Tat86 treatment suppressed β-catenin promoter activity [146]. Mutational analysis and exposure to Clade C Tat further suggest that the dicysteine motif at residues 30/31 mediated inhibition of β-catenin promoter activity [146] indicative of astrocytic activation. Astrocyte-mediated Tat neurotoxicity is has also been demonstrated in Tat86-expressing astrocytes [122] andTat101-expressing primary murine astrocytes. Tat101 expression also exerted toxicity within astrocytes including induction of cell death, oxidative stress, cytokine release and elevation of extracellular glutamate [117]. Cellular stress responses within astrocytes have also been demonstrated in Tat86 expressing astrocytes [132]. Astrocytic activation is generally associated with GFAP upregulation alongside downregulation of glutamate transporters like EAAT2, and Tat has been demonstrated to induce this phenotype (Fig. 2C). Tat-expressing astrocytes and brain tissue from PWH without encephalitis exhibit increased expression of AEG-1 which is considered to mediate EAAT2 downregulation [124]. Infection of wild-type astrocytes with HIV-1(DJV) combined with exposure to pro-inflammatory cytokines similarly enhanced AEG-1 transcripts and protein expression along with nuclear translocation. This provides a context similar to that of the latently infected CNS reservoir, where a small population of latently infected cells persists, within a milieu of chronic neuroinflammation. Nonetheless, there may be distinct pathways by which intracellular versus extracellular Tat dysregulate astrocytic glutamate homeostasis. In fetal human neuron-astrocyte co-cultures transfected to express clade B Tat, ephrin receptor A3 (EphA3) protein appears to be required for Tat-mediated downregulation of EAATs [125]. EphA3 knockdown also slightly improves the increase in extracellular glutamate observed in Tat-expressing cultures and greatly limits neuronal death. Ultimately, Tat-mediated activation of microglia and astrocytes has been well established to involve cell-specific hallmarks of activation and pathological activity, enabling both pro-inflammatory and glutamate-mediated mechanisms of neurotoxicity.

### Behavioral and Circuit Level Effects of Tat

As with in vitro models, various animal studies assessing Tat toxicity in the CNS have relied on recombinant protein to elucidate glutamatergic mechanisms of cognitive impairment. Local injection of Tat72 to hippocampus or to the ventricle is known to impair spatial learning and memory [109] – a hippocampus-dependent function – likely via NMDAR signaling [113]. Spatial learning and memory deficits are also observed long-term following neonatal exposure to recombinant Tat [152, 153]. Performance in the Morris Water Maze recapitulates that Tat *expression* in transgenic animals also impairs spatial learning and memory performance [60], emphasizing that both recombinant protein and endogenous expression drive similar impairments. These findings are supported by prior evidence that Tat potentiates NMDAR signaling, especially as NMDAR potentiation in mPFC-hippocampal circuitry is suggested to contribute to impairment in mPFC-dependent tasks [165] and NMDAR signaling in the hippocampus is required for spatial learning and memory [166]. These studies additionally suggest that Tat reduces long-term potentiation (LTP) [109, 113], offering another mechanism to examine in future studies utilizing recombinant protein and transgenic models.

One observational clinical study suggests that overall NMDAR expression is reduced in the frontal cortex of individuals with AIDS and HAD [167]. However, i*n vitro* and in vivo models provide varying results regarding the effects of Tat on NMDAR expression. These distinctions may occur based on the different extent of neuronal death, which would play a more prominent role in HAD and AIDS than what likely occurs in PWH who develop ANI and MND. Tat administration to CA1 of hippocampus was not found to alter NMDAR density [113]. However, low concentrations of recombinant Tat86 induced NMDAR-dependent synaptic loss in primary rat hippocampal neurons in the absence of overt cell death [55, 144] indicating that low levels of Tat can induce synaptic loss that occurs by sublethal mechanisms. Additional studies suggest that Tat exerts differential effects on specific NMDAR subunits both in vitro and in vivo. GluN2B specifically may mediate Tat-induced neuronal death, as GluN2B antagonism limits neuronal death [110] and can stimulate synapse replacement to rescue Tat-induced dendritic spine loss in vivo [110, 145]. This study suggests that GluN2A stimulation enables neuronal pro-survival signaling, whereas antagonism of GluN2B subunit in the presence of Tat may limit pro-death signaling. The subunit specific findings likely contribute to learning and memory symptoms, as differential recruitment of GluN2A and GluN2B affect LTP in the hippocampus [168]. Specific findings regarding NMDAR subunit involvement in learning and memory in mPFC are contradictory. Whereas hippocampus exhibits increased NR1 and NR2A expression reflective of LTP following novel environment habituation, subunit expression in mPFC is not altered [169]. However, in the context of spatial tasks, NR1 and NR2A are increased in mPFC synapses following radial maze training, followed by further NR1 increase and NR2B increases after performance in an additional test [170]. This context provides a further basis for Tat’s hippocampal effects to support spatial learning and memory impairment as observed in numerous studies, though it is important to consider region-specific changes and their effects on circuit-level function and cognition. For example, in our prior work, we have shown that recombinant Tat86 injection to PFC drove local upregulation of GluN2B transcripts in the cortex, while specifically causing spatial learning and memory impairment [59].

Characterization of the cognitive deficits observed in iTat mice indicates that Tat expression alone throughout the brain is sufficient to induce learning and memory impairment. Numerous studies have now recapitulated spatial learning and memory deficits in the iTat model [148]. This Tat-induced deficit in spatial learning and memory was accompanied by prolonged Tat-induced deficits in novel object recognition [148], suggesting that PFC and hippocampal circuitry are affected in the iTat model. By inducing Tat expression post-acquisition, further studies suggested that deficits in reversal learning actually reflect Tat-induced impairment in acquisition [140, 148]. Long-term expression of Tat in the iTat model also leads to impairment in short-term spatial learning and memory in the Morris water maze (in both male and female mice) [151], coinciding with enhanced GFAP expression in the cortex and caudate putamen and specific alteration of synaptophysin and PSD95 in female iTat mice [151]. Thus, Tat appears to exert both region- and sex-specific mechanisms of toxicity. The tetracycline-responsive aspect of the iTat model, however, exhibits leaky expression of the transgene even in the absence of induction by Dox. This feature was exploited to establish that even low-level expression of Tat, in aged animals, can lead to ventricular and dentate gyrus enlargement and synaptic loss in the absence of cytokine induction [121]. However, specific assessment of dentate gyrus revealed gliosis and IL-1β transcript upregulation in ‘leaky’ Tat animals [121]. The low-level cytokine induction and concurrent synaptic loss suggests that even a very low level of Tat expression is sufficient to promote Tat-induced gross pathology without robust inflammation. The findings of this model may inch closer toward the levels of Tat and neuroinflammation that are detected in PWH, and the effect of age in this model emphasizes its utility to reflect HAND pathogenesis. Additional models that account for the CNS cellular reservoirs in PWH and their contribution to Tat generation may help to further improve modeling of Tat’s neurocognitive effects.

### Discussion/Conclusion

Studies of varying methodology have thus indicated that HIV-1 infection and Tat exposure or expression in cells and in animals alters glutamate homeostasis; while animal models further indicate impairment in cognitive functions. The literature is highly variable in terms of Tat isoform utilized. As noted, Tat101 predominates in some populations of PWH, and isoform appears to influence specific modes of toxicity. In some cases, the results of extracellular treatment versus transfection and transgenic models suggest distinct mechanisms for glutamatergic dysregulation. Nonetheless the initial studies identifying NMDAR-mediated Tat toxicity are supported further by modulation of NMDAR expression and modulation of cognitive impairment by glutamate receptor antagonists, in intact animals and slice preparations. Tat-induced changes in glutamate transporter (GLT-1/EAAT2, xCT) expression and function have been demonstrated particularly in vitro. This dependence on in vitro models limits the translational potential of targets. Increasing use of patient-derived primary cells including iPSC-derived cultures and pursuing tractable glutamatergic therapeutic targets in in vivo systems will greatly improve the potential for novel therapeutic development for PWH experiencing HAND. Further, the investigation of Tat-induced cognitive impairment has leaned heavily on hippocampal or ventricular administration of recombinant Tat, or the iTat model. While the iTat model has revealed various cognitive impairments by Tat, it remains crucial to improve clinical relevance by understanding how Tat expression in virally-suppressed PWH on ART impacts glutamatergic signaling in multiple brain regions and contributes to neurocognitive symptoms. The iTat model has been described as using the GFAP promoter to restrict Tat expression to the CNS; nonetheless the most abundant GFAP expression is in astrocytes. Additional models express Tat within astrocytes overwhelmingly. In contrast, most Tat in PWH on ART is likely generated by infected microglia. Astrocytic expression of Tat is not considered a central cause of neuropathogenesis in the ART era. Additionally, many models of Tat neurotoxicity do not incorporate ART, which can contribute additional neurotoxicity. Further studies therefore should address: (A) expression of Tat in a smaller subpopulation of astrocytes and microglia, reflective of the estimated CNS reservoir of HIV-1; (B) distinguishing the effects of extracellular or circulating Tat versus intracellularly expressed Tat, particularly regarding actions on NMDAR, GLT-1/EAAT2 and xCT; and (C) regional and circuit-level effects of Tat in vivo, for the contribution of regional glutamate toxicity to observed cognitive impairments in animal models.

The prevention of infection via pre- and post-exposure prophylaxis, alongside ARTs, have greatly improved both lifespan and quality of life for PWH. While ARTs address multiple aspects of the viral replication cycle, the latent viral reservoir in various compartments and cell types has yet to be addressed. Current investigations regarding cure strategy focus on manipulating latency to either prevent or promote reactivation to stimulate immune clearance of infected cells. Nonetheless, various limitations on these cure strategies exist. With no functional cure, the latent reservoir persists in the CNS, enabling expression of viral transcripts and proteins including Tat; thus, specific therapeutics for HAND are still necessary to ameliorate cognitive impairment. Behavioral and cognitive approaches [171] and adjunctive therapies [172] can modify HAND symptoms. Numerous adjunctive therapies have been assessed in PWH experiencing HAND, providing limited effects [173] with ongoing tests of ART intensification [174]. More recent mechanistic studies of Tat toxicity suggest targeting endolysosomal function [175], cellular senescence [176], endocannabinoid signaling [177, 178], and excitotoxicity [179]. Candidate targets or molecules that have common roles in multiple neurocognitive diseases are of particular interest. Despite the significant portion of literature on Tat-mediated toxicity implicating enhanced glutamatergic signaling or disruption of excitatory: inhibitory balance, modulation of extracellular glutamate has not been thoroughly investigated. A brief clinical trial of memantine in AIDS dementia demonstrated the benefit of using NMDAR antagonists on neuropsychological outcomes [180] and neuronal metabolism [181], alongside significant side effects. Alternative approaches to modulating extracellular glutamate in Tat toxicity and HAND are important to offer improved efficacy and safety. The preclinical development of novel modulators of glutamate transporters offers a targeted therapy to improve both indirect and direct mechanisms of toxicity as observed with Tat. As some glutamatergic effects of Tat are also observed with other HIV-1 proteins, the development and preclinical testing of novel therapeutics targeting glutamate toxicity are relevant for treatment of HAND. Alternative approaches to modulating extracellular glutamate in also have potential benefit in other neurodegenerative diseases including Parkinson’s, Alzheimer’s and other neurodegenerative diseases [182, 183].

## Key References

- Ye, X., Y. Zhang, Q. Xu, H. Zheng, X. Wu, J. Qiu, et al. (2017) HIV-1 Tat inhibits EAAT-2 through AEG-1 upregulation in models of HIV-associated neurocognitive disorder. Oncotarget. 8(24): p. 39,922–39,934.
  - ○ This paper established disruption of the synaptic glutamate transporter EAAT2 by Tat exposure, highlighting an important mechanism for Tat-induced astrocytic glutamate toxicity.
- Mastrantonio, R., V. D’Ezio, M. Colasanti, and T. Persichini (2019) Nrf2-Mediated System x(c)(-) Activation in Astroglial Cells Is Involved in HIV-1 Tat-Induced Neurotoxicity. Mol Neurobiol. 56(5): p. 3796–3806.
  - ○ This paper extends the understanding of Tat-mediated microglial activation and its contribution to neurotoxicity, particularly by modulation of the cystine-glutamate antiporter.
- Nass, S.R., Y.K. Hahn, V.D. McLane, N.B. Varshneya, M.I. Damaj, P.E. Knapp, et al. (2020) Chronic HIV-1 Tat exposure alters anterior cingulate cortico-basal ganglia-thalamocortical synaptic circuitry, associated behavioral control, and immune regulation in male mice. Brain Behav Immun Health. 5.
  - ○ This publication demonstrates circuit-level alterations, highlighting region-specific changes that affect excitatory: inhibitory balance and behavior in Tat-transgenic mice.
- Ewens, A.N., A. Pilski, S.D. Hastings, C. Krook-Magnuson, S.M. Graves, E. Krook-Magnuson, et al. (2024) Levetiracetam Prevents Neurophysiological Changes and Preserves Cognitive Function in the Human Immunodeficiency Virus (HIV)-1 Transactivator of Transcription Transgenic Mouse Model of HIV-Associated Neurocognitive Disorder. J Pharmacol Exp Ther. 391(1): p. 104–118.
  - ○ This publication emphasizes the central role of elevated glutamatergic transmission in learning and memory impairment in Tat-transgenic mice.
- Duffy BC, King KM, Nepal B, Nonnemacher MR, Kortagere S. Acute Administration of HIV-1 Tat Protein Drives Glutamatergic Alterations in a Rodent Model of HIV-Associated Neurocognitive Disorders. Mol Neurobiol. 2024 Oct;61(10):8467–8480. doi: https://doi.org/10.1007/s12035-024-04113-8. Epub 2024 Mar 22. PMID: 38,514,527; PMCID: PMC11415472.
  - ○ This paper demonstrates that Tat drives differential expression in glutamatergic circuitry suggesting molecular correlates of Tat-induced learning and memory impairment.

## Data Availability

No datasets were generated or analysed during the current study.
